# Laparoscopic management for stump appendicitis: A case series with literature review

**DOI:** 10.1097/MD.0000000000018072

**Published:** 2019-11-22

**Authors:** Hanlim Choi, Young Jin Choi, Taek-Gu Lee, Dae Hoon Kim, Jae-Woon Choi, Dong Hee Ryu

**Affiliations:** a Department of Surgery, Chungbuk National University Hospital; b Department of Surgery, Chungbuk National University College of Medicine, Cheongju, Korea.

**Keywords:** laparoscopy, stump appendicitis, appendectomy

## Abstract

**Introduction:**

Appendectomy is one of the most common emergency surgical operations. Stump appendicitis is a rare complication after appendectomy and is caused by acute inflammation of the remnant part of the appendix. Because of the low index of suspicion owing to a previous history of appendectomy, the diagnosis of stump appendicitis is often delayed.

**Methods:**

Between January 2008 and December 2017, 6 patients were diagnosed with stump appendicitis with or without perforation at a single institution. They had undergone operative management with laparoscopic approach. The clinical data of these patients were retrospectively analyzed by reviewing the medical records and pathologic reports.

**Results:**

Five patients were male, with a mean age of 42.4 years (range 11–77 years). The time interval after initial appendectomy ranged from 2 weeks to 30 years. Three patients underwent laparoscopic completion appendectomy, and the others underwent laparoscopic ileocecectomy. The mean hospital stay was 9 days (range 5–13 days). There were no cases of open conversion.

**Conclusions:**

Stump appendicitis is a rare complication after appendectomy. A laparoscopic procedure can be performed for management of stump appendicitis with or without perforation.

## 1 Introduction

An appendectomy is one of the most common emergency surgical operations.^[1]^ Stump appendicitis is a rare complication after appendectomy and is caused by acute inflammation of the remnant portion of the appendix.^[2]^ Because of both the low incidence of stump appendicitis and a low index of suspicion owing to a previous appendectomy, the diagnosis of stump appendicitis is often delayed. Also, delayed diagnosis makes surgery more difficult and increases morbidity. Here, we describe 6 cases of stump appendicitis which were treated through laparoscopic procedures, successfully.

## 2 Methods

From January 2008 to December 2017, 6 patients were diagnosed as having a stump appendicitis with or without perforation, and they underwent laparoscopic procedures at the Department of Surgery at Chungbuk National University Hospital, Korea. All patients were admitted to the emergency room with right abdominal pain and assessed using contrast-enhanced abdominal and pelvic computed tomography (A-P CT). We retrospectively reviewed the patients’ medical records and radiologic images. Retrospective protocol of this study was approved by the institutional review board (IRB) of Chungbuk National University Hospital, South Korea (IRB No 2019-02-006-001). Informed written consent was obtained from the patients for publication of this case report and accompanying images.

## 3 Results

**Figure 1:**
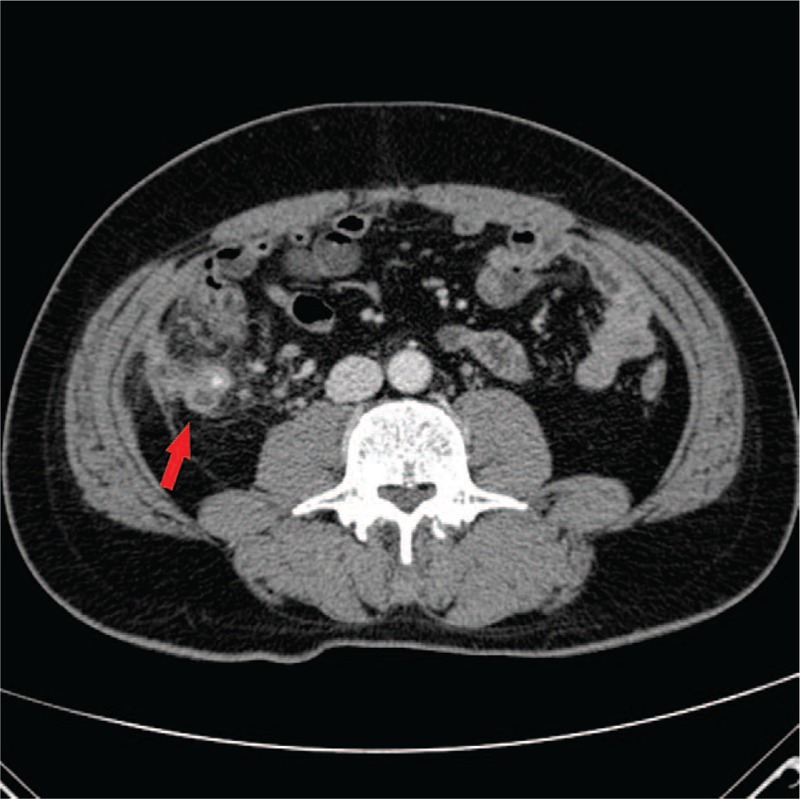
Contrast-enhanced abdominal and pelvic computed tomography in case 1. An appendicolith in the short stump appendix with inflammation around the appendix (arrow).

**Figure 2:**
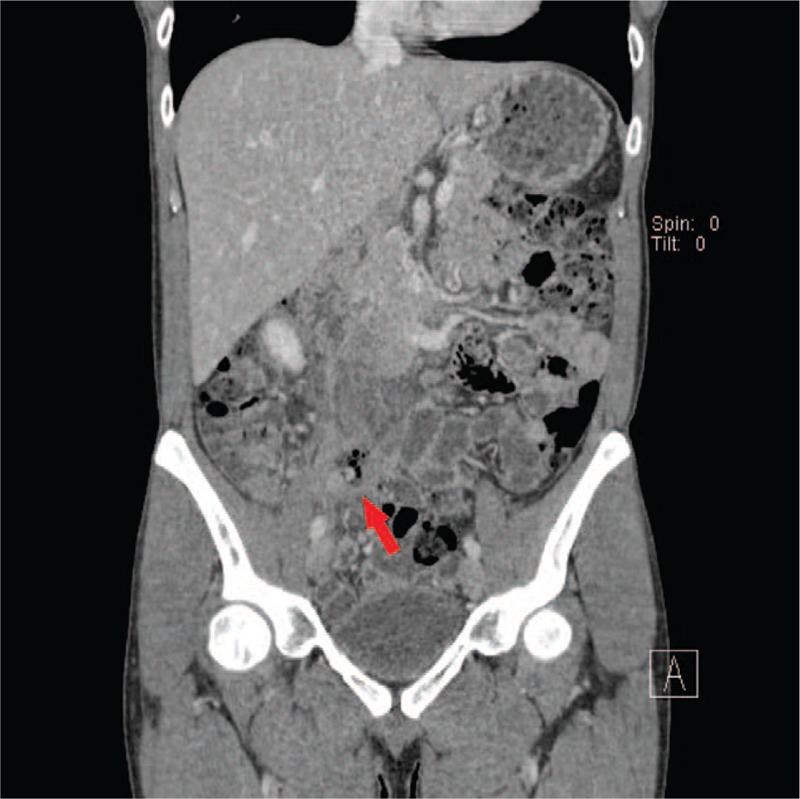
Contrast-enhanced abdominal and pelvic computed tomography in case 4. A perforation of the tip of stump appendix (arrow).

**Figure 3:**
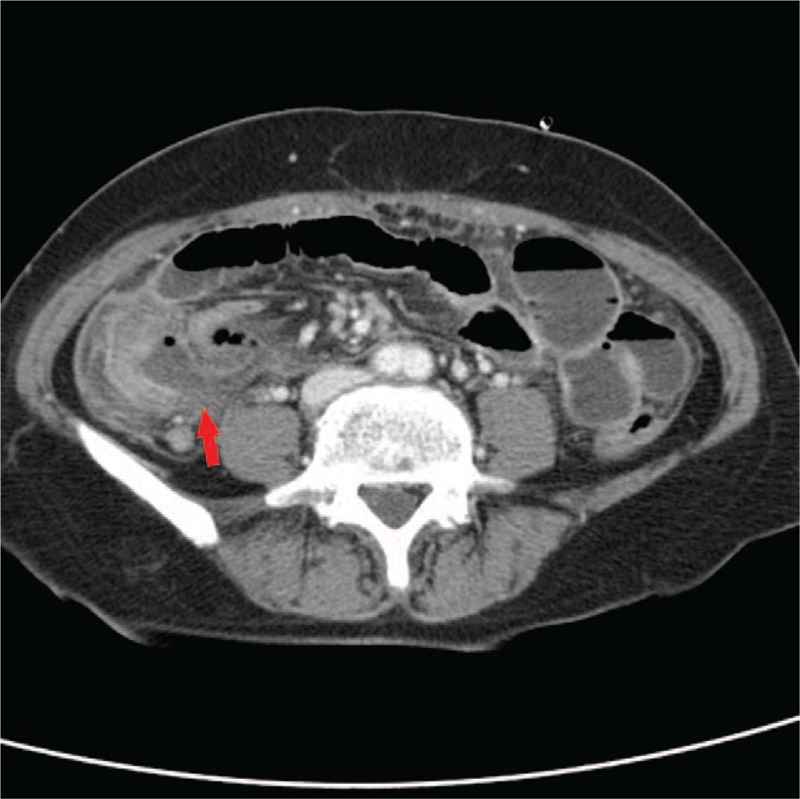
Contrast-enhanced abdominal and pelvic computed tomography in case 5. A pericecal inflammatory infiltration with abscess and bowel edema (arrow).

Five patients were male, with a mean age of 42.4 years (range 11–77 years). The mean time from initial appendectomy to presentation was 17.3 months (range 2 weeks–30 years). Four patients had previously undergone an open appendectomy. On A-P CT scans, 3 patients demonstrated perforation around the stump appendix. In cases 1 and 3, there was an appendicolith in the short stump appendix with inflammation around the appendix (Fig. 1). In case 4, A-P CT scans showed perforation of the stump appendiceal tip (Fig. 2). These 3 patients underwent laparoscopic completion appendectomy. In cases 2, 5, and 6, A-P CT scans found a pericecal inflammatory infiltration with abscess and bowel edema (Fig. 3). These 3 patients underwent laparoscopic ileocecectomy. By the pathologic reports, the mean stump length was 2.3 cm (range 1–4 cm) (Table 1). The mean hospital stay was 9 days (range 5–13 days). All patients were discharged in good general condition.

**Table 1:**
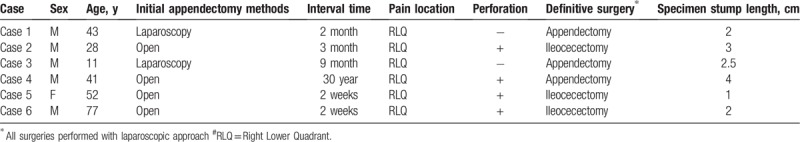
Characteristics of 6 patients of stump appendicitis.

## 4 Discussion

The first stump appendicitis was described by Rose in 1945, and Subramanian and Liang analyzed 61 cases that were reported in 2012.^[1,3]^ A stump appendicitis is caused by acute inflammation of the remnant of the appendix. Roberts et al^[4]^ reported that the interval time after initial appendectomy was ranged from 4 days to 40 years. Although the symptoms and signs are very similar to those of acute appendicitis, the diagnosis of stump appendicitis is often delayed because of the low index of suspicion owing to the previous appendectomy.

The causes of a stump appendicitis are associated with surgical factors related to the previous appendectomy. The most common factor is a long stump of appendix due to inadequate confirmation of the appendicular base during surgery.^[5,6]^ The recommended stump size which prevents stump appendicitis is <5 mm, generally.^[7,8]^ Most recently, Roberts et al recommended a stump size of <3 mm.^[4]^

Contrast-enhanced A-P CT scanning is useful in the diagnosis of stump appendicitis, including remnants of appendiceal luminal dilatation, pericecal inflammatory infiltration, and abscess formation.^[9]^ O’Leary et al^[10]^ reported that the incidence of perforated stump appendicitis was about 70%.

Following the introduction of laparoscopic appendectomy, there have been reports comparing the incidence of stump appendicitis in laparoscopic and open approaches.^[11,12]^ In 2010, Society of Gastrointestinal and Endoscopic Surgeons(SAGES) presented the guidelines for laparoscopic appendectomy.^[13]^ Subramanian and Liang^[1]^ reported a lower incidence of stump appendicitis in the laparoscopic than in the open appendectomy.

The treatment of choice for stump appendicitis is a completion appendectomy, even when perforation has occurred. However, in cases of severe inflammation with abscesses, an ileocecectomy is needed. A laparoscopic approach can be used for management of stump appendicitis with or without perforation. And we have performed laparoscopic procedures in all our cases without surgical complications.

## 5 Conclusions

Although a stump appendicitis is a rare complication after an appendectomy, it can be a cause of right-sided abdominal pain in patients who have had appendectomies previously. Because delayed diagnosis is associated with increased postoperative morbidity and also affects the extent of resection, clinicians should consider the possibility of stump appendicitis as a cause of acute abdominal pain. A laparoscopic approach can be adopted for management of stump appendicitis with or without perforation.

## Acknowledgment

The authors thank Editage (www.editage.com) for English language editing.

## Author contributions

**Conceptualization:** Hanlim Choi, Dong Hee Ryu.

**Data curation:** Hanlim Choi, Dae Hoon Kim.

**Investigation:** Dae Hoon Kim.

**Supervision:** Jae-Woon Choi, Dong Hee Ryu.

**Writing – original draft:** Hanlim Choi.

**Writing – review & editing:** Young Jin Choi, Taek-Gu Lee, Dong Hee Ryu.
